# Identification of EML4-ALK as an alternative fusion gene in epithelioid inflammatory myofibroblastic sarcoma

**DOI:** 10.1186/s13023-017-0647-8

**Published:** 2017-05-23

**Authors:** Quan Jiang, Han-Xing Tong, Ying-Yong Hou, Yong Zhang, Jing-Lei Li, Yu-Hong Zhou, Jing Xu, Jiong-Yuan Wang, Wei-Qi Lu

**Affiliations:** 1 Departments of General Surgery, Zhongshan Hospital, Fudan University, Shanghai, 200032 China; 2 Departments of Oncology, Zhongshan Hospital, Fudan University, Shanghai, 200032 China; 3 Departments of Pathology, Zhongshan Hospital, Fudan University, Shanghai, 200032 China

**Keywords:** Inflammatory myofibroblastic tumors (IMTs), Epithelioid inflammatory myofibroblastic sarcoma (EIMS), *EML4-ALK*, *RANBP2-ALK*, RNAseq

## Abstract

**Background:**

Known as solid tumors of intermediate malignant potential, most inflammatory myofibroblastic tumors (IMTs) are treatable as long as the tumor is en-bloc resected. However, in some cases, the tumors have recurred and grown rapidly after successful surgery. Some of these tumors were classified as an epithelioid inflammatory myofibroblastic sarcoma (EIMS). Most previously reported EIMSs have been caused by *RANBP2-ALK* fusion gene. We herein report an EIMS case caused by an *EML4-ALK* fusion gene.

**Methods:**

RNAseq was conducted to find out the new *ALK* fusion gene which could not be detected following previously reported RT-PCR methods for EIMS cases with *RANBP2-ALK* fusion gene. After that, RT-PCR was also conducted to further prove the newly found fusion gene. Immunohistochemistry (IHC) and fluorescence in situ hybridization (FISH) test were applied to find out the unique morphological characters compared with the previous reported EIMS cases.

**Results:**

We found an EIMS case who was suffering from a rapid recurrence after cytoreducyive surgery was done to relieve the exacerbating symptoms. The patient finally died for tumor lysis syndrome after the application of crizotinib. Distinctive ALK staining under the membrane and relatively weak ALK staining in the cytoplasm could also be observed. RNAseq and RT-PCR further revealed that the tumor harbored an EML4-ALK fusion gene.

**Conclusion:**

In conclusion, this is the first EIMS demonstrated to have been caused by the formation of an *EML4-ALK* fusion gene. This enriches the spectrum of EIMS and enlarges the horizon for the study of EIMS. The experience we shared in managing this kind of disease by discussing aspects of its success and failure could be of great value for surgeons and pathologists.

**Electronic supplementary material:**

The online version of this article (doi:10.1186/s13023-017-0647-8) contains supplementary material, which is available to authorized users.

## Background

Once known as inflammatory pseudotumors or inflammatory fibrosarcomas, inflammatory myofibroblastic tumors (IMTs) are rare mesenchymal tumors composed of myofibroblastic spindle cells admixed with lymphocytes, plasma cells and eosinophils [1]. IMTs usually present as a solid mass originating from the lung and abdominopelvic region and have a tendency to affect children and young adults of both genders [1, 2]. Once treated as reactive inflammatory lesions, IMTs are better known now as solid tumors with intermediate malignant potential. Though most IMTs can be treated by en bloc resection, the recurrence rate remains high, ranging from <2 to 25% in pulmonary and extrapulmonary sites, respectively, and metastasis occurs in <5% of cases, [1, 2] indicating that these unique cases are worth intensive study.

Anaplastic lymphoma kinase (ALK), also known as ALK tyrosine kinase receptor or CD246 (cluster of differentiation 246), is an enzyme that in humans is encoded by the *ALK* gene. ALK plays an important role in the development of the brain and exerts its effects on specific neurons in the nervous system [3, 4]. As an oncogenic driver, aberrant *ALK* has been recognized as the central trigger of the development of a number of different tumor types, including hematopoietic, epithelial, mesenchymal and neural neoplasms [5]. About 50% of IMTs harbor an *ALK* gene rearrangement, accompanied by positive immunohistochemistry (IHC) staining of ALK protein [6]. As the first *ALK* fusion gene found, *NPM-ALK* was first reported by Morris and colleagues in anaplastic large cell lymphomas harboring a t(2; 5) translocation [3]. More *ALK* fusion genes were subsequently reported in various tumors. Though it still remains controversial how the fusion genes cause tumors., some common features have been revealed The rearrangement of partner genes of *ALK* could provide active promoters to *ALK* and lead to the overexpression of the fusion genes. The N-terminal sequence encoded by the nonreceptor tyrosine kinase member of the pair replaces the extracellular and transmembrane domains of ALK and contributes protein-binding sites that allow oligomerization and mimicking of ligand binding, ultimately leading to constitutive, ligand-independent ALK autophosphorylation and activation [6–8]. For IMTs, partner genes reported have included *NPM* [3], *TPM3* [7], *TPM4* [7], *CLTC* [9], *RANBP2* [10], *CARS* [11], *ATIC* [12], *SEC31L1* [13]*, EML4* [14]*, TFG* [15]*, LMNA* [16], *FN1* [16],*PPFIBP2* [17], *DCTN1* [18] and *RRBP1* [19]. *RANBP2-ALK*-associated EIMS has attracted the most attention for its unique pathological and clinical manifestations. RAN binding protein 2 (RANBP2) is a small GTP-binding protein of the RAS superfamily that is mainly located in the nuclear membrane [2]. Patients with IMT harboring RANBP2-ALK have been reported to suffer from rapid local recurrences and a higher death rate. The pathology features were characterized by epithelioid cells with a nuclear membrane or perinuclear ALK staining pattern. Based on their distinctive biological behavior, *RANBP2-ALK* associated IMTs have been categorized as epithelioid inflammatory myofibroblastic sarcoma (EIMS) [6].

Echinoderm microtubule-associated protein-like 4 (EML4) is a protein participating in mitotic nuclear division and other microtubule-based processes. It is distributed along with microtubules in the cytoplasm and membranes [20]. Initially found in non-small cell lung cancer (NSCLC), *EML4-ALK* fusion has been regarded as an important event in the development of lung cancer and an indication for the application of crizotinib (an ALK inhibitor) [16]. Both being located on the short arm of chromosome 2, *EML4* and *ALK* are rearranged by a paracentric inversion of the region [inv(2)(p21p23)] [21]. The *EML4-ALK* fusion was once thought to occur only in lung cancer. However, recent studies of 8 IMT cases with *EML4-ALK* fusions have revealed that they could also play a role in the development of IMTs [14, 16]. However, the clinical features of *EML4-ALK* associated IMTs remain unclear.

As mentioned above, some IMT cases could still show rapid recurrence following en bloc resection. EIMS typically represent this type of IMT. However, some patients without a *RANBP2-ALK* fusion gene have similar clinical complaints. We recently found an *EML4-ALK* associated IMTs with clinical and pathological manifestation matching the diagnosis criteria of EIMS. The goal of our study is to raise awareness of the possible link between *EML4-ALK* fusion genes and aggravated clinical manifestation and to put forward the opinion that crizotinib is effective, but vigilance is needed in the treatment of this kind of tumor.

## Case report

**Fig. 1:**
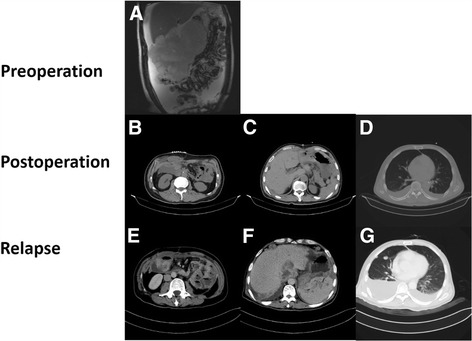
**a**. Preoperative abdominal MRI demonstrated a solid mass with an irregular lobulated configuration located in the right upper quadrant with a diameter of 20 cm (*blue arrow*). **c, e, g**. Patient was suffering from rapid recurrence with an extensive metastatic lesion in the abdominal cavity. **b, d, f**. The tumor was radically resected and the corresponding CT radiograph showed no lesions

A 45-year-old man, without significant personal or familial medical history, was admitted with a history of abdominal distention and intermittent abdominal pain for a month. Abdominal MRI was conducted to identify a solid mass with an irregular lobulated configuration located in the right upper quadrant with a diameter of 20 cm. (Figure 1a). Biopsy and pathology examinations came to no conclusion. The patient had no hematemesis or melena and no elevated lesions on the skin. Physical examination indicated that a huge mass could be palpated with tenderness in the upper quadrant. With only symptomatic treatment, the patient still complained of an exacerbation of abdominal distention and reduction of urine. Bloody fluid was observed in catheter drainage. Cystometry was then conducted to conform a diagnosis of abdominal compartment syndrome. Evidence indicated that the patient could be suffering from a tumor rupture and hemorrhage. Surgery was performed to relieve the symptoms and resect the tumor.

**Fig. 2:**
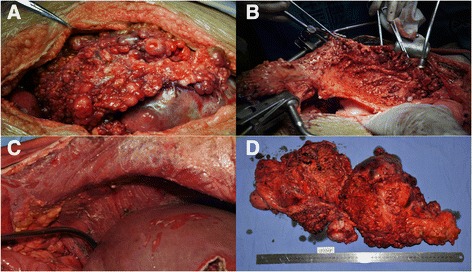
**a**. The peritoneum and the surface of the mesenterium were both implanted with numerous nodules whose diameter varied from 0.5 to 3 cm. **b**, **c**. Total peritoneal resection was performed to dissect the implanted lesions on the peritoneal tissue. **d**. Lesions that were visible were smoothly resected

Laparotomy indicated that the enterocelia was filled with ascites with a drainage of 5000 ml. The major part of the tumor was located in the right upper quadrant, invading the transverse colon, omentum and gastroduodenum. The peritoneum and the surface of the mesenterium were both implanted with numerous nodules with a diameter that varied from 0.5 to 3 cm. (Figure 2a) In the paracolic ditch of both sides, the right phrenic top and pelvic area were all affected by tumors. A cytoreductive surgery was then performed to remove as much of the visible tumor as possible. The surgeries performed included total peritoneal resection, partial hepatectomy, subtotal colectomy, partial small bowel resection, partial right diaphragm resection and terminal ileostomy. (Figure 2b, c, d) The surgery went smoothly with 1200 ml blood loss. A CT scan on September 21 showed the tumors had been totally resected and left no obvious metastatic lesions (Fig. 1b, d, f).

The pathological test revealed that the tumor was composed of both epithelioid and spindle cells with intermediate grade nuclear atypia and a mitotic count of 11/50 HPF. An IHC assay revealed that both spindle and epithelioid cells displayed positive cytoplasmic staining of ALK, SMA and desmin. Florescence in situ hybridization was also conducted to test for *ALK* rearrangement. This primary pathology finding provided a convincing diagnosis of inflammatory myofibroblastic tumor. Considering the tumor was spreading all over the abdominal cavity and that ALK staining of the tumor was positive, crizotinib was suggested as adjuvant therapy. However, the patient was unable to tolerate treatment with crizotinib because he was suffering from severe vomiting. The elevation of ALT and AST of serum also indicated liver damage. We had to stop treatment temporarily.

A month after the operation, the patient began to complain of abdominal pain and uncontrollable vomiting. The vomitus was an acid liquid with a volume of 500 ml daily. No excreta could be seen in the pouch. A solid mass with a diameter of 5 cm could also be palpated around the ileostomy. The patient was diagnosed with incomplete intestinal obstruction. A CT scan was conducted and revealed tumors recurring with extensive metastasis involving the liver, spleen, small intestine and right pleural cavity (Fig. 1c, e, g). Bilateral pleural and abdominal effusions could also be observed.

Crizotinib remained to be the only option to save the patient’s life. However, following the rapid progression of intestinal obstruction, the administration of crizotinib seemed impossible because of the unstoppable vomiting. Administration through the nasogastric tube was also performed, but turned out to be ineffective because the drug was thrown up for severe vomiting. Finally, we came up with a possible solution for administration of crizotinib—giving the drug by nasogastric tube through the terminal ileal stoma created during the operation. After getting consent from the Ethical Committee of Zhongshan Hospital and the patient, we started treatment by giving crizotinib 100 mg bid. The dose was gradually increased to 200 mg bid based on the monitoring blood tests. The capsule was opened and the powder was mixed with pure water. The mixture was then injected into the nasogastric tube which had been placed backward into the end ileum with a length of 50 cm from the stoma. After one week’s administration, the patient presented with less vomiting and reflux and was even able to consume solid food. Excreta could be seen in the pouch. The evaluation of prealbumin also indicated an improvement of nutritional level. Compared with the previous treatment, the patient was suffering less from vomiting but elevation of ALT was still observed. However, after two weeks’ treatment with the drug, oliguria suddenly appeared. Further blood and urine tests indicated hyperuricemia (1071 μmol/L) and an abnormal creatinine level (440 μmol/L), indicating the patient’s kidneys had shut down. Based on the patient’s symptoms and auxiliary examinations, the diagnosis of tumor lysis syndrome (TLS) was confirmed. We immediately gave dialysis and other auxiliary treatment. However, the disease progressed so fast that the patient suffered cardiac arrest and was not able to be revived.

## Methods

This study protocol was approved by the Ethical Committee of Zhongshan Hospital. After informed consent, tissue samples were obtained from the patient.

### Immunohistochemistry (IHC)

Formalin-fixed paraffin-embedded sections (5 μm thick) were immunostained for ALK,SMA and desmin. Briefly, after deparaffinization and antigen retrieval, the slides were incubated with anti-ALK (5A4; 1:100; citrate buffer pressure cooker retrieval; Leica Biosystems, Buffalo Grove, IL, USA) anti-SMA (SAB5500002,1:50, citrate buffer pressure cooker retrieval; Sigma,IL,USA), and anti-Desmin (ab32362,1:100, citrate buffer pressure cooker retrieval,Abcom, Cambridge, UN) overnight at 4 °C. The slides were then washed and detected with the REAL EnVision Detection System, Peroxidase/DAB+, Rabbit/Mouse (DAKO, #K5007).

### Fluorescence in situ hybridization (FISH)

Fluorescence in situ hybridization (FISH) was carried out on 4 μm thick paraffin sections according to the manufacturer’s instructions. *ALK* rearrangement was determined using the Vysis LSI ALK dual color break apart probe (Abbott Molecular, Des Plaines, IL USA).

### Method for RNAseq

mRNA library construction strictly followed the manual of the TruSeq Stranded mRNA Library Prep Kit. (Vazyme Biotech, IL USA) The mRNA was purified and fragmented. Then cDNA was synthesized from the mRNA fragments by random primers. The cDNA fragments of the constructed libraries were hybridized to the surface of flow cells and amplified to form clusters, and then sequenced with the Illumina HiSeq X sequencing system.

First, these sequencing reads were mapped to the human (*Homo sapiens*) genome (version hg19) and transcriptome (gencode v19) using RNA STAR software (v2.4.0), which can align reads across splice junctions with or without gene annotations. Then STAR-Fusion (v0.4.0) was used to detect gene fusions based on the alignment results. To evaluate gene expression, we estimated transcript abundance with featureCounts (v1.4.6) using gene annotations from the gencode v19 database. FPKM values were calculated with R package edgeR (v3.8.5).

### PCR analysis of *EML4-ALK* fusion

Reverse transcription polymerase chain reaction (RT-PCR) was performed on the total RNA extracted from 5 mm^3^ fresh tissue using the RecoverAll Total Nucleic Acid Isolation Kit (Ambion/Applied Biosystems, Austin, TX USA) according to the manufacturer’s instructions. Reverse transcription was set up on 1 μg of total RNA with random hexamer primers and SuperScript III reverse transcriptase (Invitrogen, Carlsbad, CA USA). Three primer pairs (Primer pair 1: F 5′-CAAAGCAGTAGTTGGGGTTG-3′; R 5′-ACCAAAACTGCAGACAAGCA-3′, Primer pair 2: F 5′-ACTGATGGAGGAGGTCTTGC-3; R 5′-ACCAAAACTGCAGACAAGCA-3′, Primer pair 3: F 5′-GTCTTGCCAGCAAAGCAGT-3; R 5′-ACCAAAACTGCAGACAAGCA-3′) were designed for PCR tests as follows: 94 °C for 45 s, 60 °C for 30 s, and 72 °C for 90 s. PCR products were directly sequenced using forward and reverse PCR primers. Hg19 was the human genomic build used.

## Results

**Fig. 3:**
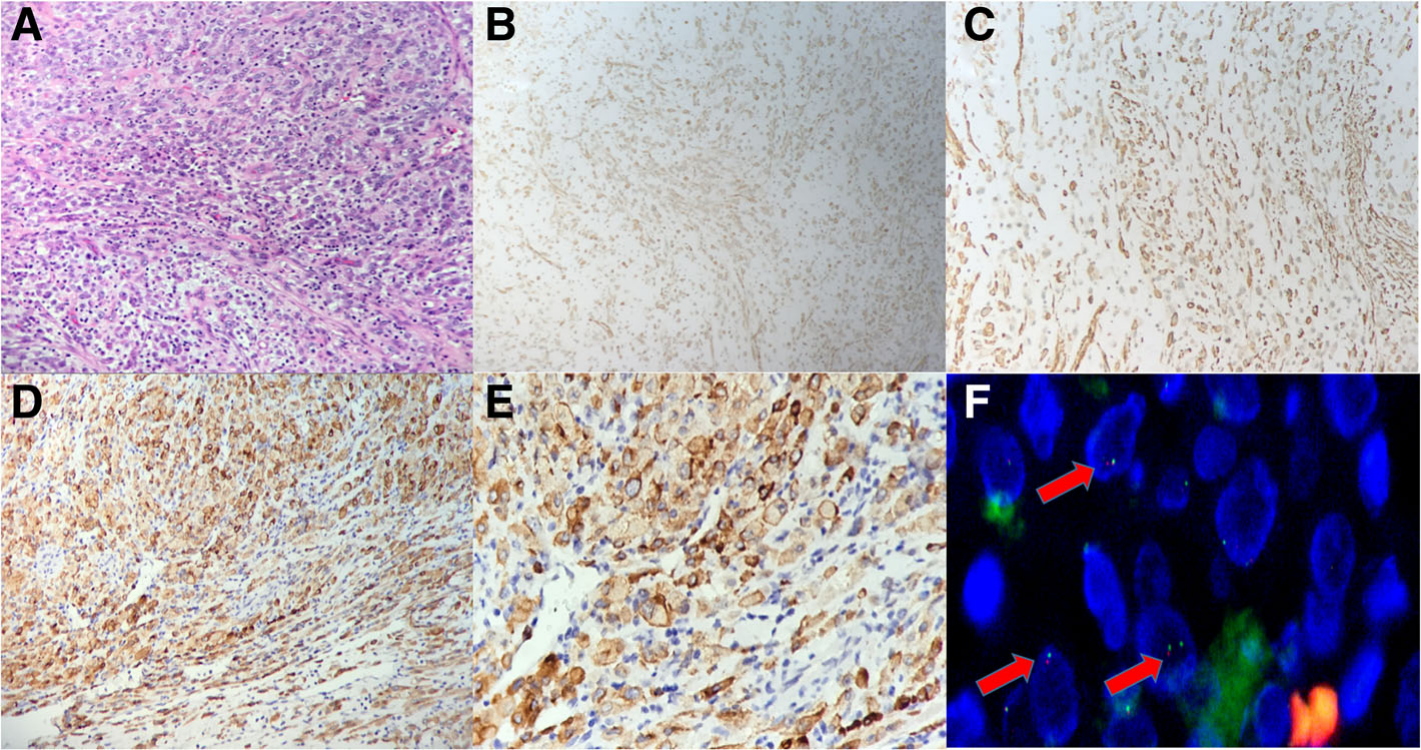
**a.** Tumors were composed of both large epithelioid cells and spindle cells. **b, c, d**. The tumor cells were positive for SMA (B), desmin (C) and ALK (D). **e.** IHC for ALK showed positive staining in both large epithelioid cells and spindle cells. The staining was most distinctive under the membranes. **f.** Fluorescence in situ hybridization showing splitting of the two signals corresponding to the 3′ and 5′ ends of ALK, confirming the presence of an *ALK* rearrangement

Morphologically, the tumors were equally dominated by epithelioid and spindle cells with prominent infiltrating neutrophils. The stroma was mainly composed of collagenous tissue. (Figure 3a) IHC for SMA and Desmin were positive in both epithelioid and spindle cells. (Figure 3b, c) Besides, IHC for ALK showed cytoplasmic and cell membrane staining for both epithelioid and spindle cells. The most distinctive site for ALK staining was the cytoplasm under the membrane, which was consistent with the distribution of EML4 in the cell. (Figure 3d, e) The phenomenon was different from previously reported *RANBP2-ALK* associated IMTs, in which ALK staining was located in the nuclear membrane. Fluorescence in situ hybridization showed splitting of the two signals corresponding to the 3′ and 5′ ends of ALK, confirming the presence of an ALK rearrangement (red arrows). (Figure 3f) All the tests above revealed that this was an EIMS case.

Before conducting RNAseq, we performed RT-PCR to test for the presence of a *RANBP2-ALK* fusion gene. However, we were unable to confirm the existence of this fusion gene even when repeating the test with different primers for *RANBP2*. We conducted RNAseq to clarify the situation, which revealed that the tumor harbored an *EML4-ALK* fusion gene (Additional file 1).

**Fig. 4:**
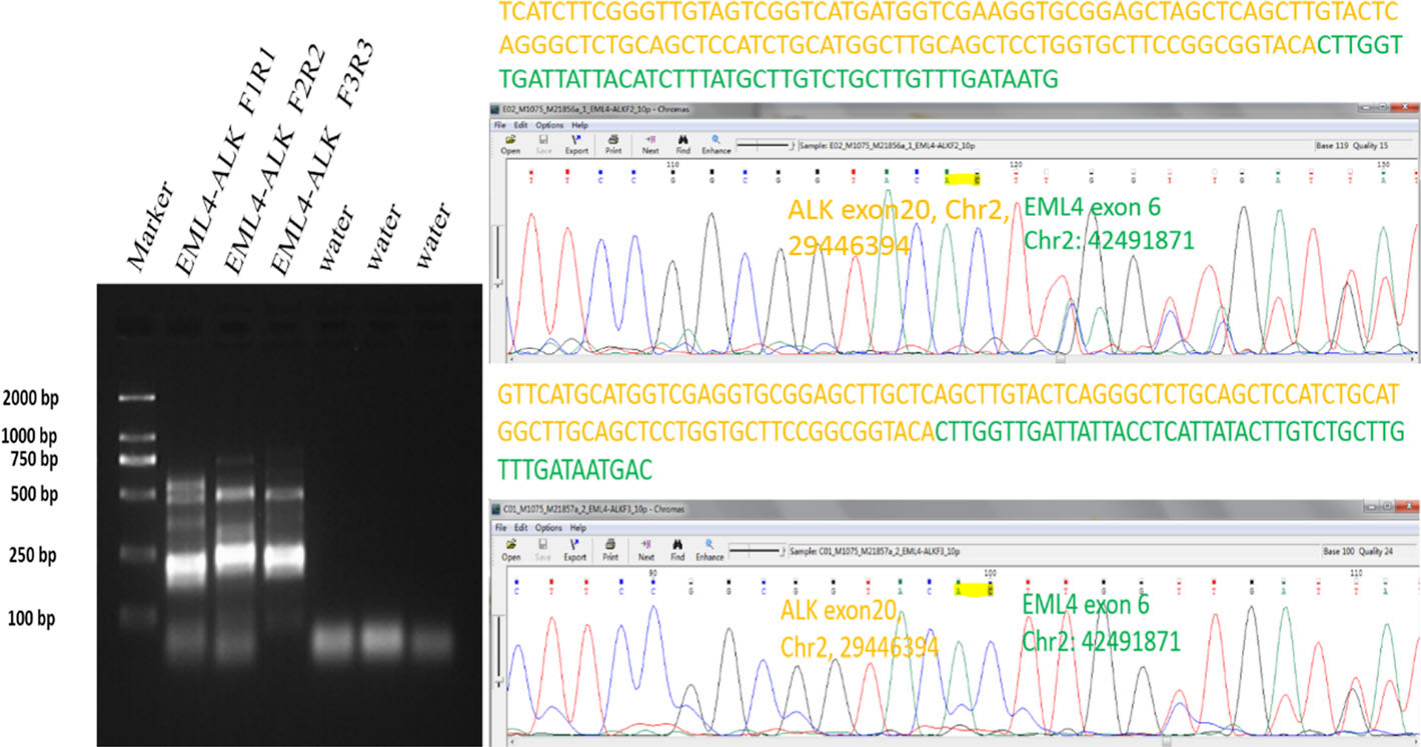
**a.** PCR products of 3 primer pairs. The length of the products was 199 bp (Primer pair 1), 222 bp (Primer pair 2) and 209 bp (Primer pair 3). **b, c.** Direct sequencing of the products of Primer pairs 2 and 3 confirmed an *EML4-ALK* fusion gene detected by RNAseq. The product of Primer pair 1 did not reveal the presence of a fusion

To confirm the RNAseq results, RT-PCR was performed based on three primer pairs. The results from Primer pairs 2 and 3 validated the *EML4-ALK* fusion and its breakpoints (Fig. 4).

## Discussion

EIMS was first named and characterized in 2011 [6]. Though the concept was newly raised, potential EIMS could have been observed in previous studies. Cook and colleagues reported that in 73 IMT patients, some presented with more aggressive biological behaviors and were predominated by round cells instead of spindle cells [10]. Chen and colleagues described the similar phenomenon by reporting an IMT’with round cell transformation’ harboring RANBP2-ALK [22]. In the study putting forward this new subtype, EIMS was described as a highly aggressive IMT variant with epithelioid-to-round cell morphology, vesicular nuclei, large nucleoli, amphiphilic-eosinophilic cytoplasm, and nuclear membrane or perinuclear ALK staining. This kind of tumor is usually intraabdominal and malignant. For IHC, tumors were all positive for ALK, and mostly showed a nuclear membrane staining pattern [23]. Some studies have also raised diagnostic criteria including: 1) round-to-epithelioid tumor cells; 2) abundant myxoid stroma with inflammatory infiltrate; 3) immunopositivity for ALK [24]. However, the criteria remain to be validated because they only give a diagnosis based on phenotypic characters, without specific objective indicators. Despite that, these are still the most commonly acknowledged criteria. Although RANBP2-ALK is by far the most common fusion gene responsible for EIMS, the identification of EML4-ALK in the current EIMS case, as well as the RRBP1-ALK fusion in a recent report [19], justified the exclusion of RANBP2-ALK fusion from the diagnostic criteria of EIMS.

*EML4-ALK* fusion genes have attracted great attention from oncologists focusing on lung cancer since two related landmark studies were published. Though harbored by only 4–6% of lung adenocarcinomas, *EML4-ALK* fusion has been recognized as the second most important event to consider in the targeted treatment of lung cancer, following the EGFR mutation [25, 26]. According to data derived from studies of lung cancer, *EML4-ALK* fusion occurs through a paracentric inversion within the short arm of chromosome 2, where EML4 and ALK genes are both located. ALK is a member of the insulin receptor kinase superfamily, composed of a transmembrane helix, a cytoplasmic tyrosine kinase domain and an extracellular region that includes two MAM (meprin, A5 protein and protein tyrosine phosphatase mu) domains [5, 8, 20]. The breakpoint of the *ALK* gene lies close to the 5′ end of exon 20. Thus, the products of the *EML4-ALK* fusion gene only include the ALK’s intracellular region. However, the break points of the *EML4* gene are more variable. At least 15 variants have been reported [19]. Previous studies have demonstrated that the oncogenesis pathways of the ALK-associated fusion genes are mediated mainly by the ALK part, with the fusion partners providing active promoters and dimerization/oligomerization mechanisms to activate the ALK tyrosine kinase domain. In addition, the fusion partners could also localize the fusion proteins to specific subcellular compartments, indicating that the locations of the proteins as demonstrated by IHC tests could be suggestive of the identity of the partners [5, 20].

*EML4-ALK* fusion genes have not been linked with EIMS. 8 IMT cases harboring an *EML4-ALK* fusion gene have been discovered in two previous studies, but the clinical manifestations did not match the diagnosis of EIMS [14, 16]. However, after carefully reviewing one of the previous studies, we discovered one potential case originating from the lung was found containing epithelioid cells and the patient was suffering from a rapid recurrence after en bloc resection [14]. This may further support a possible link between *EML4-ALK* fusion genes and EIMS.

Intestinal obstruction is one of the most common complications for aggressive recurring tumors [27]. Considering the absorptive function of the gastrointestinal tract is weakened under conditions of obstruction, prescribing crizotinib orally is sometimes impossible. An intravenous formulation is still not available. As stated in the previous section, the patient was suffering from severe vomiting caused by an intestinal obstruction after the tumor recurred. This left us no choice but giving the drug by a nasogastric tube through the ileal stoma. This is an unconventional way of feeding that has never been reported before. Through that procedure, the drug was actually placed only in the terminal 50 cm of the ileum, so the absorptive area for crizotinib was only that region. Based on the package insert for crizotinib and recent literature, we noted that the specific site of absorption in the gastrointestinal tract is unknown, and specific pharmacokinetic studies have not formally evaluated the method of capsule disintegration and administration via a stomach tube. Though it is an isolated case, our experience provides a hint that the terminal ileum could be an important place for crizotinib absorption and EIMS patients harboring EML4-ALK fusion genes could be sensitive to crizotinib.

As a group of metabolic abnormalities caused by the disintegration of malignant cells following the instigation of chemotherapy, TLS tends to affect patients with high tumor burden, especially those with hematological disease. However, TLS is rarely seen in solid tumors [28]. Reviewing our case, the patient was suffering from hyperuricemia (1071 μmol/L) and increased creatinine (440 μmol/L) after the initially successful treatment with crizotinib. The occurrence of TLS matched the rapid progression of the tumor and the efficiency of application of crizotinib. The clinical management of TLS relies greatly on prevention, and early intervention is indispensable to achieve better outcomes [29, 30]. Therefore, we recommend to allocate every EIMS patient to appropriate risk groups evaluated according to the recent TLS risk classification before beginning chemotherapy [29]. In order to remove the massive excess of uric acid and protect renal function, all patients require increased fluid intake and close monitoring of fluid output. Monitoring of plasma uric acid, creatinine, potassium, phosphate and calcium are as essential as the strict assessment of fluid input and output [28]. If TLS occurs, the final step of treatment is considered to be renal dialysis. Peritoneal dialysis, hemodialysis and various other forms of dialysis have been used in the treatment of TLS, and all appear to be effective and can be expected to rapidly address fluid overload and reverse biochemical abnormalities [31]. Dialysis must be continued for several weeks until urine output and renal function have recovered considerably Administering dialysis treatment at the early stage in the clinical course of TLS may improve the outcome in patients with multiorgan failure [32]. Though it is currently unknown whether cutting the dose would be helpful to prevent TLS, we suggest reconsidering the administration dose of crizotinib on the basis of the tumor burden.

## Conclusion

In conclusion, this is the first EIMS demonstrated to have been caused by the formation of an *EML4-ALK* fusion gene. This enriches the spectrum of EIMS and enlarges the horizon for the study of EIMS. The experience we shared in managing this kind of disease by discussing aspects of its success and failure could be of great value for surgeons and pathologists.

## Additional file

Additional file 1: The original data of RNAseq. (XLSX 19961 kb)

## Abbreviations

ALK: Anaplastic lymphoma kinase
CD246: Cluster of differentiation 246
EIMS: Epithelioid inflammatory myofibroblastic sarcoma
EML4: Echinoderm microtubule-associated protein-like 4
FISH: Fluorescence in situ hybridization
IHC: Immunohistochemistry
IMTs: Inflammatory myofibroblastic tumors
NSCLC: Non-small cell lung cancer
RANBP2: RAN binding protein 2
RT-PCR: Reverse transcription polymerase chain reaction
TLS: Tumor lysis syndrome
